# *Wolbachia* Mediate Variation of Host Immunocompetence

**DOI:** 10.1371/journal.pone.0003286

**Published:** 2008-09-26

**Authors:** Christine Braquart-Varnier, Marion Lachat, Juline Herbinière, Monique Johnson, Yves Caubet, Didier Bouchon, Mathieu Sicard

**Affiliations:** Université de Poitiers, Laboratoire Ecologie, Evolution, Symbiose, UMR CNRS 6556, 40 avenue du recteur Pineau, Poitiers, France; University of Birmingham, United Kingdom

## Abstract

**Background:**

After decades during which endosymbionts were considered as silent in their hosts, in particular concerning the immune system, recent studies have revealed the contrary. In the present paper, we addressed the effect of *Wolbachia*, the most prevalent endosymbiont in arthropods, on host immunocompetence. To this end, we chose the *A. vulgare*-*Wolbachia* symbiosis as a model system because it leads to compare consequences of two *Wolbachia* strains (*w*VulC and *w*VulM) on hosts from the same population. Moreover, *A. vulgare* is the only host-species in which *Wolbachia* have been directly observed within haemocytes which are responsible for both humoral and cellular immune responses.

**Methodology/Principal Findings:**

We sampled gravid females from the same population that were either asymbiotic, infected with *w*VulC, or infected with *w*VulM. The offspring from these females were tested and it was revealed that individuals harbouring *w*VulC exhibited: (i) lower haemocyte densities, (ii) more intense septicaemia in their haemolymph and (iii) a reduced lifespan as compared to individuals habouring *w*VulM or asymbiotic ones. Therefore, individuals in this population of *A. vulgare* appeared to suffer more from *w*VulC than from *w*VulM. Symbiotic titer and location in the haemocytes did not differ for the two *Wolbachia* strains showing that these two parameters were not responsible for differences observed in their extended phenotypes in *A. vulgare*.

**Conclusion/Significance:**

The two *Wolbachia* strains infecting *A. vulgare* in the same population induced variation in immunocompetence and survival of their hosts. Such variation should highly influence the dynamics of this host-symbiont system. We propose in accordance with previous population genetic works, that *w*VulM is a local strain that has attenuated its virulence through a long term adaptation process towards local *A. vulgare* genotypes whereas *w*VulC, which is a widespread and invasive strain, is not locally adapted.

## Introduction

Investigations on the consequences of endosymbionts on their host's fitness have revealed that some of them exhibit variable effects, blurring the distinction between mutualism and parasitism. It is now admitted that symbionts are deeply involved in the evolutionary process of their hosts and that variations in symbiont genotypes may trigger more important differences in host life history traits than variations of the host genotypes themselves [1]. Thus, the entity that undergoes selection is clearly the extended phenotype of symbionts in their host. Within such a conceptual framework, the studies that focus on an understanding of host population dynamics need to consider symbiosis as a central parameter. Of the endosymbionts known to exhibit various effects on their host fitness, *Wolbachia* are the most prevalent in arthropods. The diversity of the interactions between *Wolbachia* and their hosts is mainly illustrated by the various strategies these endosymbionts exhibit in order to secure their vertical transmission. Hence, in some host species *Wolbachia* decrease the fitness of uninfected individuals (i.e. cytoplasmic incompatibility) while in others they increase female ratio in populations (i.e. parthenogenesis, male-killing and feminization) [2], [3]. As the transmission of *Wolbachia* is vertical, their fitness is directly linked to the fitness of their hosts. Therefore, such situations could be seen as favourable to evolve towards obligate symbiosis and therefore mutualism. However, such obligate *Wolbachia* symbioses have only been described in filarial nematodes and in the parasitic wasp *Asobara tabida* [4], [5]. In both models, aposymbiotic females failed to produce mature oocytes showing that *Wolbachia* are obligate for reproduction [4], [5]. Other studies have shown that *Wolbachia* may be mutualists by improving development, survival and reproduction of their hosts [6]–[10]. However, these effects can vary over time or with respect to host genotypes and cause continuous evolutionary changes [7], [10].

Despite these few examples of dependency or mutualism between *Wolbachia* and their hosts, *Wolbachia* are mostly described as facultative endosymbionts that negatively influence their hosts' life history traits, including body size [11], fecundity [11]–[13], survival [13]–[15], larval competitiveness [16], male fertility and sperm cyst production [17] and mating choice [18]. Recently, Fytrou et al. (2006) [19] hypothesized that *Wolbachia* may also immunodepress their hosts. Indeed, they observed that *Drosophila* infected by *Wolbachia* showed less encapsulation of parasitic wasp eggs than cured ones. The capacity of *Wolbachia* to interact with the arthropod immune system has also been recently suggested by (i) the discovery of an intracellular sensor of Gram (-) bacteria in *Drosophila* and (ii) the observed modifications of the immune response in *Drosophila melanogaster* and *Aedes albopictus* cell lineages due to the presence of *Wolbachia* [20]–[22]. Moreover, molecular evolution studies within *Wolbachia-*infecting insects have revealed that the *Wolbachia* outer membrane protein *wsp,* which has been shown to play a role in filarial nematode infection success [23], is under strong positive selection thus suggesting that invertebrate immune response may be an important selection factor for *Wolbachia* [24]. In invertebrates, immune cells (i.e. haemocytes) are essential effectors of immunity [20]. They are responsible for both cellular (encapsulation, phagocytosis etc.) and humoral (antimicrobial peptides, phenoloxydase cascade etc.) responses. *Wolbachia* have been observed within host haemocytes in only one species: the terrestrial isopod *Armadillidium vulgare* [25]. In light of this observation and also due to the even more central role of haemocytes in crustacean immune systems [26], the *A. vulgare-Wolbachia* association appears to be a very pertinent biological model to study the influence of symbiosis on host immunocompetence. In addition, *A. vulgare* is of particular interest because individuals from the same population are mono-infected (to date, no naturally co-infected individuals have been observed) by one of three different strains of *Wolbachia* (*w*VulM, *w*VulC and *w*VulP) [27], [28]. *w*VulP, which has only been identified recently and which is less prevalent than the others, seems to be the result of a recombination event between *w*VulM and *w*VulC suggesting that co-infections do occur but are unstable. This would explain why co-infected individuals are never observed in sampled populations [28]. Both *w*VulC or *w*VulM are feminizing strains since offspring from sampled *A. vulgare* females are highly female-biased [27]. *A. vulgare* lineages infected with *w*VulC or *w*VulM are maintained in laboratory for decades showing that the symbiotic transmission and feminizing phenotypes of these two *Wolbachia* strains persist through generations (Bouchon et al., unpublished data). However, despite these similarities, population genetic studies suggest that *w*Vul strains exhibit different strategies [27], [29]. *w*VulC would be the most invading strain able to replace previous *Wolbachia* strains including *w*VulM [27], [29], whereas *w*VulM would be the resident strain, more locally adapted to host genotypes. As immunocompetence is obviously a primordial parameter in host dynamics, it is thus of great interest to compare the effect of these two *Wolbachia* strains on host immune capacities.

In the present study, a comparison was made of the extended phenotype of *w*VulC and *w*VulM in *A. vulgare.* To this end, we evaluated in *A. vulgare* individuals infected by *w*VulC or *w*VulM: (i) the titer of *Wolbachia* in ovaries by qPCR (ii) the presence of *Wolbachia* in haemocytes by electronic microscopy, (iii) the density of haemocytes, (iv) the intensity of natural septicaemia (i.e. number of CFU obtained from haemolymph) (v) their survival over a 7 month period. These results were used to detect differences in immunocompetence and survival of *A. vulgare* as a function of *Wolbachia* genotype.

## Materials and Methods

### *A. vulgare* lineages

Gravid females (F0) of *A. vulgare* were sampled in the natural park of Chizé (Western France 46°08′05″N-0°24′21″W) and brought back to the laboratory. The infection status of each gravid female (infected with *Wolbachia w*VulC or *w*VulM or asymbiotic i.e. noninfected by *Wolbachia*) was determined as described below. To avoid any maternal effect in further experiments at least three gravid females (F0) for each infection status were used to start lineages. Their offspring (F1) were born and reared in the laboratory. For each infection status, one hundred virgin females from the F1 generation were kept and placed individually with one male (F1) in individual boxes. Over a 7 month period, the survival of these F1 females was monitored every three days and their progenies (F2) were collected. The virgin F2 females grew during two years before immunocompetence experiments. In order to simplify the reading of the paper, virgin asymbiotic females were called A females, virgin females infected with *w*VulC were called C females, virgin females infected with *w*VuM were called M females. All of these lineages were grown at 20°C on moistened potting mix derived from peat from sphagnum moss (pH = 6.4 and conductivity = 50.0 mS/m) with dead leaves and carrot slices as a food source.

Additionally, a lineage of females experimentally infected by *w*VulC (herein called injC females) was created. For this, the ovaries of 10 C females (F0) were collected and crushed into 1ml of Ringer solution. The resulting suspension was filtered through a 1.2 µm pore membrane, and 1 µl of filtrate injected into non gravid A females (F0) using a thin glass needle (Bouchon et al., 1998). F0 injC females were then crossed with asymbiotic males for two generations in order to produce two year old F2 virgin injC females.

F2 females (A, C, M) were used to assess: *Wolbachia* titer, haemocytes density and intensity of natural septicaemia. Due to a small number of individuals, injC females (F2) were only used to highlight the assessment of the effect of *w*VulC on haemocyte density.

### Infection status and *Wolbachia* titer

The infection status of each *A. vulgare* female used in experiments was determined by a PCR-RFLP assay. Individuals were dissected and total DNA extracted from the ovary as previously described [30]. PCR amplifications were then performed to test for presence/absence of *Wolbachia* using specific primer sets for the *wsp* gene [31] and conditions as previously described [27]. In order to discriminate each *Wolbachia* strain, a PCR-RFLP test was performed based on the analysis of *w*VulC and *w*VulM *wsp* sequences (*w*VulM: AJ419984 and *w*VulC: AJ419987). Two restriction enzymes were used: *Bsr*I, which cuts *wsp* amplicons in *w*VulC but not in *w*VulM, and *MFe*I, which cuts *wsp* amplicons in *w*VulM but not in *w*VulC.

Comparative analysis of the titer of *Wolbachia* in ovaries between C females and M females was performed using qPCR of the *wsp* gene. Total DNA from the ovaries of 20 females of each infection status were individually extracted [30]. To prepare the standard, 7 µl of purified *wsp* gene PCR product were directly ligated into a pGEM-T-easy vector (Promega) and one site was cut with *Nc*ol enzyme at 37°C overnight to linearize the plasmid. Plasmid concentration was subsequently determined using a spectrophotometer and the number of *wsp* copies calculated. For each DNA sample, the qPCR was carried out under the following conditions: 2 µl of 10× Light Cycler Mix (Roche™), 0.2 µl of 20 µM of *wsp* primers, 1.6 µl of 25mM MgCl2. The thermal cycling used an initial denaturation period of 8 min at 95°C, followed by 45 cycles of denaturing temperature at 95°C for 15 sec., the annealing temperature for the reaction was 57°C for 14 sec. and 72°C for 28°C and a final extension step at 72°C for 28 sec.

### Haemolymph sampling

Haemolymph was sampled in the same way for all following experiments: cuticles were disinfected by immersing individuals for 30 sec. in a 10% sodium hypochlorite solution followed by a 30 sec. immersion in distilled water. The cuticle was then pierced dorsally between the sixth and seventh abdominal segments using a fine needle and 10 µl haemolymph were collected with a micropipette.

### *Wolbachia* in haemocytes

The haemolymph from 20 females of each infection status (A females, C females and M females) was individually sampled and half diluted in an anticoagulant solution [Modified Alsever's solution MAS 27 mM sodium citrate: 336 mM NaCl, 115 mM glucose, 9 mM EDTA, pH 7; [32]]. Haemocytes were separated from plasma by centrifugation (400× g, 10 min, 4°C) and washed with the same buffer. Haemocytes were fixed (9% glutaraldehyde, 0.3M sodium cacodylate, 3% NaCl, v/v/v) for 45 min at 4°C and then centrifuged (400× g, 10 min, 4°C). Cells were washed (0.3M sodium cacodylate, 3% NaCl, 0.8M sucrose, v/v/v) for 15 min at 4°C then centrifuged (400× g, 10 min, 4°C). Haemocytes were included in a 2% agar gel (37°C) and 1mm^3^ plugs were cut and placed in wash buffer for 2h at 4°C following which they were post fixed into 4% OsO_4_, 0.3M sodium cacodylate, 5.5% NaCl for 45 min. Haemocytes were subsequently dehydrated through a graded series of acetone solutions, infiltrated, and embedded in resin (Spurr, Polyscience Inc.). Thick sections (0.5 µm) were stained with 1% toluidin blue. Thin sections (90nm) were contrasted by incubation in 1% uranyl acetate in 50% ethanol for 1 min, and then stained with lead citrate. Sections were observed using a transmission electronic microscope (JEOL 100C).

### Haemocyte density in haemolymph

The haemolymph (10 µl) of 35 females of each infection status (A females, C females, M females and injC females) was individually sampled and added to 10 µl of MAS and 60 µl of 0.4% Trypan blue to discriminate dead haemocytes from living ones. The actual number of living haemocytes in each sample was evaluated using a Thoma counting chamber.

### Natural septicaemia assessment

The haemolymph (10 µl) of 60 females of each infection status (A females, C females and M females) was individually sampled and added to 290 µl of LB medium. An aliquot of 100 µl of this suspension was streaked onto one plate of each of the three different solid agar media used: (i) a non selective chocolate medium (Biomérieux) on which most bacteria, even fastidious ones, can grow, (ii) the Columbia Nalidixic Acid Agar (CNA) (Biomérieux) in order to preferentially select Gram (+) bacteria and (iii) the Mueller-Hinton Agar (MHA) (Biomérieux) (35g/l) with 10% sheep's blood and 10 µl/l vancomycin in order to preferentially select Gram (−) bacteria. After 3 days at 28°C, the number of colony forming units (CFUs) on each plate was determined.

### Statistical analyses

All statistical analyses were performed using JUMP software (JMP, 2001, ver.4.03; SAS Institute, Cary, NC, USA). Survival estimates were assessed by a Kaplan-Meier analysis followed by a univariate Survival Analysis using a Wilcoxon test. As haemocyte density and natural septicaemia data showed homoscedasticity of variance (Levene test *p*>0.05), difference in mean responses was tested by an ANOVA followed by PLSD Fisher post-hoc test.

## Results

### Infection status and *Wolbachia* titer

**Figure 1:**
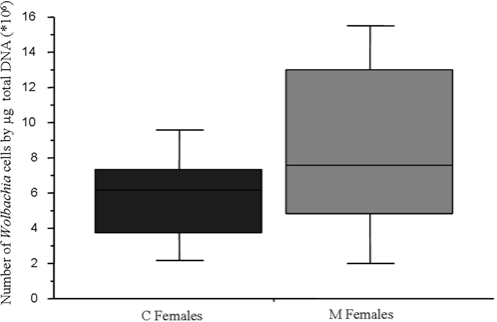
Titer of each *Wolbachia* strains in *A. vulgare* ovaries. Comparative analysis of the titer of *Wolbachia* in ovaries between C females and M females was performed using qPCR of the *wsp* gene. The two strains of *Wolbachia* exhibited a similar titer (∼7,640×10^6^ bacteria per µg total DNA; ANOVA, *F*_1,35_ = 1.92, *p* = 0.17).

All C, injC and M females used in this experiment were controlled positive for *Wolbachia*. The two strains of *Wolbachia* exhibited a similar titer (∼7,640×10^6^ bacteria per µg total DNA) in the host (ANOVA, *F*_1,35_ = 1.92, *p* = 0.17). However, *w*VulM tended to show higher titer than *w*VulC (Fig. 1).

### Presence of *Wolbachia* in haemocytes

**Figure 2:**
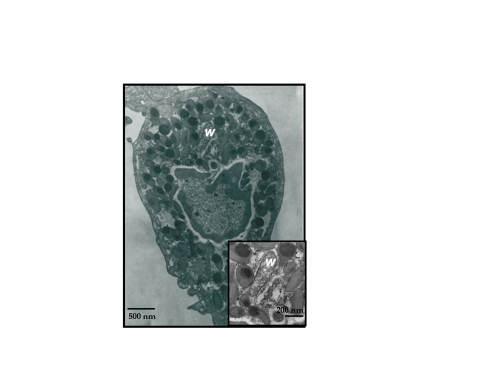
Haemocyte from an *A. vulgare* female infected with *w*VulC observed by transmission electronic microscopy. Haemocytes were included in agar gel and cut. Thick sections (0.5 µm) were stained and observed using a transmission electronic microscope.*Wolbachia* (notated *w* on the photography) cells were observed by transmission electronic microscopy in haemocytes of all C and M females tested.

*Wolbachia* cells were observed by transmission electronic microscopy in haemocytes of all C and M females tested. In haemocytes, *Wolbachia* were included in a vacuole and did not seem to be undergoing any type of degradation process suggesting that they may survive and perhaps even multiply within such cells (Fig. 2).

### Effect of *Wolbachia* on host survival

**Figure 3:**
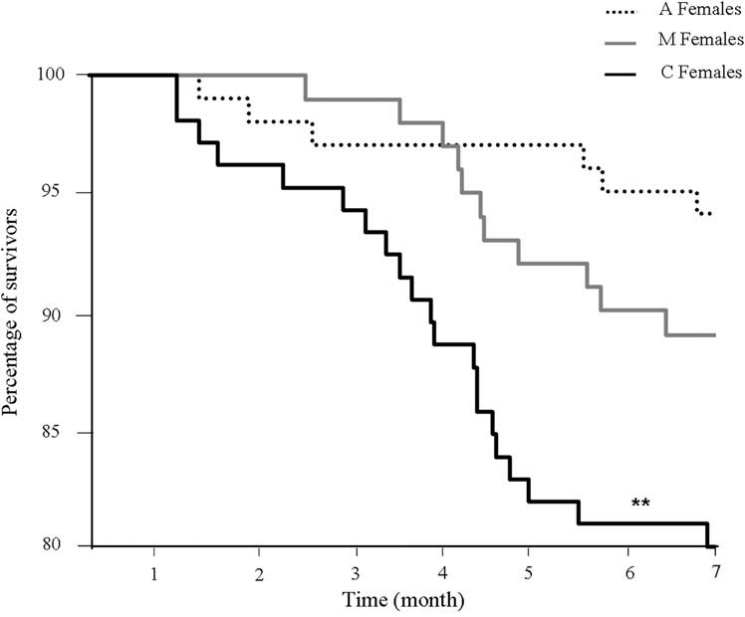
Effect of *Wolbachia* on host survival. Comparison of survival plots between A females, C females and M females during 7 months revealed that C females survived significantly less than A females and M females.

Comparison of survival plots between A females, C females and M females revealed significant differences (Wilcoxon test, χ^2^ = 10.87 *df* = 2 *p* = 0.004). C Females survived significantly less (19% mortality, mean time before death = 177.4±4.4 days) than A females (6% mortality, mean time before death = 189.1±2.7 days) (Wilcoxon test, χ^2^ = 9.39 *df* = 1 *p* = 0.002) whereas survival of M females (11% mortality, mean time before death = 177.8±2.1 days) was not significantly different from that of A females (Wilcoxon test, χ^2^ = 1.56 *df* = 1 *p* = 0.212) (Fig. 3). Finally, C females survived significantly less than M females (Wilcoxon test, χ^2^ = 4.05 *df* = 1 *p* = 0.044).

### Effect of *Wolbachia* on haemocyte density

**Figure 4:**
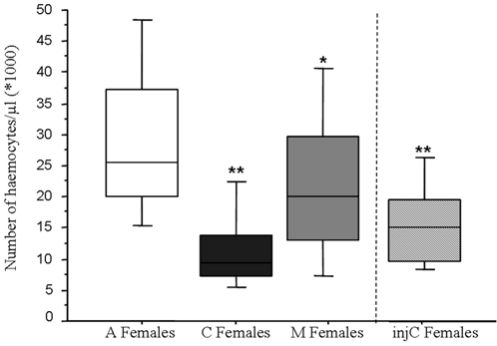
Effect of *Wolbachia* on haemocyte density. Global comparison of haemocyte densities in haemolymph of *A. vulgare* females infected or not by *Wolbachia* revealed that A females exhibited significantly higher haemocyte densities than C females, M females and injC females.

Global comparison of haemocyte densities in A females, C females, M females and injC females exhibited significant heterogeneity (ANOVA, *F*_3,141_ = 18.91, *p*<0.0001) (Fig. 3). Statistical analysis revealed that A females exhibited significantly higher haemocyte densities (mean: 29,731 haemocytes per µl) than (i) C females (mean: 11,760 haemocytes per µl) (Fisher's PLSD test: *p*<0.0001), (ii) M females (mean: 22,805 haemocytes per µl) (Fisher's PLSD test: *p* = 0.0232) and injC females (mean: 15,722 haemocytes per µl) (Fisher's PLSD test: *p*<0.0001). However, M females exhibited higher haemocyte densities than either C females (Fisher's PLSD test: *p*<0.0001) or injC females (Fisher's PLSD test: *p*<0.009) (Fig. 4). C females and injC females showed similar haemocyte densities (Fisher's PLSD test: *p* = 0.104).

### Effect of *Wolbachia* on natural septicaemia

**Figure 5:**
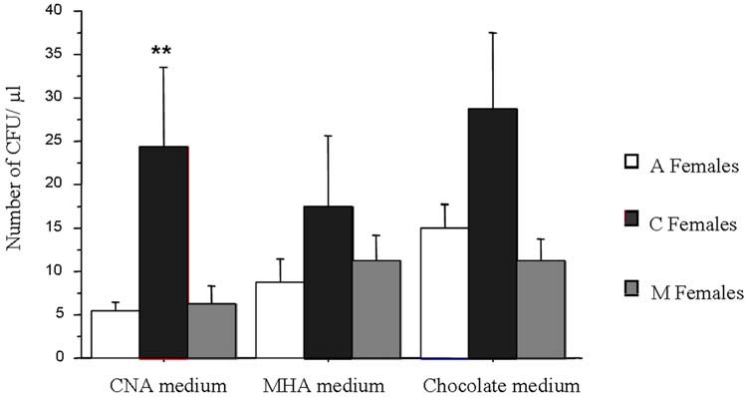
Effect of *Wolbachia* on CFUs obtained from haemolymph samples. Haemolymph samples from *A. vulgare* females infected or not by *Wolbachia* were streaked onto several agar media (CNA, MHA and Chocolate). On CNA [selective medium for Gram (+) bacteria], the mean number of CFUs obtained for haemolymph samples from C females was significantly higher than in A females or M females.

On CNA [selective medium for Gram (+) bacteria], the mean number of CFUs obtained for haemolymph samples from (i) A females, (ii) C females and (iii) M females showed heterogeneity (ANOVA, *F*_2,174_ = 3.961, *p* = 0.0208). The mean CFUs was significantly higher in the haemolymph from C females (mean: 81 bacteria/µl) than in A females (mean: 18 bacteria/µl) (Fisher's PLSD test: *p* = 0.0133) or M females (mean: 21 bacteria/µl) (Fisher's PLSD test: *p* = 0.0179). Differences between mean CFUs on MHA [selective medium for Gram (-) bacteria)] and chocolate medium (“non-selective” medium) were not significant (ANOVA, *F*_2,174_ = 0.769, *p* = 0.4650 and *F*_2,174_ = 2.850, *p* = 0.0606, respectively) (Fig. 5). However, in all media, C females tend to harbour more bacteria in their haemolymph than other females (Fig. 4).

## Discussion

Infection dynamics of vertically transmitted endosymbiotic bacteria is highly dependant on their host's reproductive success. However, symbionts such as *Wolbachia* often lead to physiological alterations which can negatively impact host fitness. Among potentially important effects, the impact on immunocompetence seems of particular interest in light of its fundamental role on host fitness. Two arthropod bacterial endosymbionts (*Serratia symbiotica* and *Hamiltonella defensa*) have been demonstrated to increase pea aphid resistance towards parasitoids showing that some vertically transmitted symbionts are able to improve immunocompetence [1], [33]. For *Wolbachia*, which are the most frequent endosymbiotic bacteria in arthropods, two recent studies suggest that they may interact with the host immune system and thus modify the host's ability to overcome infection by other parasites [19], [22]. In the present study, we showed that reduction of haemocyte density in *A. vulgare* was due to *Wolbachia* and not to difference in host genotypes. Such reduction is an indication of immunodepression as haemocyte load is a determinant factor in the ability of crustaceans to mount an efficient immune response against parasites [26]. However, differences in the effects of the two *Wolbachia* strains on *A. vulgare* were observed: C females had less haemocytes but also more intense septicaemia than M females. These results highly suggest that haemocyte density and intensity of septicaemia are linked and that, in *A*. *vulgare, w*VulC is a more important immunodepressing biotic factor than *w*VulM.

Three non exclusive hypotheses can be proposed to explain how *w*VulC triggers a decrease in haemocyte density. A first hypothesis involves a direct negative effect of *Wolbachia* on haemocytes survival *via* toxins. Such /*Wolbachia*/-toxins could for example interfere with apoptosis in haemocytes as previously described for other cell types [34], [35]. A second hypothesis is that the decrease in haemocyte density is due to the impact of *Wolbachia* load in haemocytes whereby high symbiotic densities in a cell would lead to its destruction as previously described in other tissues for *w*Pop [36]. This hypothesis is further supported by previous studies in which *w*VulC has been shown to generate effects comparable to those of *w*Pop when injected into foreign recipient hosts [3], [37]. However, haemocytes observed here by transmission electronic microscopy mainly exhibited low bacterial loads. A third hypothesis would be that the global physiological cost of *Wolbachia* on their hosts leads to a decrease in their immunocompetence due to a drop in haemocyte production and a reduced capacity to cure bacteria from the haemolymph.

Our data revealed that the differences in immunocompetence and survival, in the population of *A. vulgare* we studied, are due to *Wolbachia* strains they harbour. The strain *w*VulC was the most immunodepressing and also reduced host lifespan the most, suggesting that these two life history traits may be linked and showing that *w*VulC is clearly more virulent than *w*VulM. This difference in virulence between *w*VulC and *w*VulM seems not due to different titer or location in haemocytes between these two *Wolbachia* strains but can be interpreted in the light of population genetic works conducted on the same populations [27], [29]. Such studies showed that the *w*VulC strain was widely distributed and associated with all *A. vulgare* mitochondrial lineages while *w*VulM was restricted to particular host mitochondrial lineages. In the area where gravid females (i.e. F0) were sampled, the mitochondrial lineages associated with *w*VulM are very frequent [27], [29]. Taking into account these data, Cordaux et al. (2004) proposed a scenario in which *w*VulM is a locally adapted strain (i.e. resident) while *w*VulC is invasive and widely distributed all over the world. Such a scenario, associated with expected evolutionary trends, would suggest that local adaptation occurred between *w*VulM and local host genotypes leading to the observed attenuation of its virulence compared to *w*VulC.

Even if virulence seems to decrease during local adaptation processes, we have demonstrated here that *Wolbachia* symbiosis is costly and can lead to a reduced lifespan for *A. vulgare.* It is hard to understand selective forces which would promote and maintain such genomic conflicts between symbionts and their hosts in the context of vertically transmitted symbioses. This discrepancy can be seen as the consequence of various strategies adopted by symbionts in order to invade host populations. While symbionts such as those in the pea aphid may spread by increasing their host's immunocompetence [1], [33], *Wolbachia* rely on manipulating host reproduction which can generate indirect costs. Such costs would tend to keep the prevalence of *Wolbachia* at lower levels than those predicted by feminizing effects alone and could help explain the low frequency of symbiotic females observed in natural populations of *A. vulgare* [29].
